# *Candida auris* colonization or infection in the ear of four patients with chronic otitis externa: A case series and literature review

**DOI:** 10.22034/cmm.2025.345406.1658

**Published:** 2026-02-01

**Authors:** Fatemeh Safari, Shima Aboutalebian, Tooraj Khaledian, Elahe Nasri, Kazem Ahmadikia

**Affiliations:** 1 Department of Microbiology, Falavarjan Branch, Islamic Azad University, Isfahan, Iran; 2 Department of Medical Parasitology and Mycology, School of Medicine, Isfahan University of Medical Sciences, Isfahan, Iran; 3 Department of Otolaryngology, School of Medicine, Al-Zahra Hospital, Isfahan University of Medical Sciences, Isfahan, Iran; 4 Infectious Diseases and Tropical Medicine Research Center, Isfahan University of Medical Sciences, Isfahan, Iran; 5 Department of Medical Parasitology and Mycology, School of Public Health, Tehran University of Medical Sciences, Tehran, Iran

**Keywords:** *Candida auris*, Clade V, Iran, Otomycosis, Species-specific PCR

## Abstract

**Background and Purpose:**

*Candida auris*, a globally emerging multidrug-resistant pathogen, was first isolated from the external ear canal of a Japanese patient. Subsequently, most cases were reported from invasive or non-ear infections; however, in Iran, an increasing number of otologic infections have been reported as the dominant clinical form of *C. auris* infections.

**Materials and Methods:**

This study described the clinical features, microbiological findings, and outcome of four *C. auris* otomycotic cases that had been identified during a previous large-scale survey performed by the authors on 1,029 patients/specimens in a single center. It is accompanied by a literature review of *C. auris* cases reported from Iran up to 2023.

**Results:**

Six culture-proven *C. auris* cases were reported, of which four belonged to clade V and two to clade I. Three culture-negative, polymerase chain reaction-identified cases were also documented. The clinical profile of the infection in Iran showed a distribution of *C. auris* clades distinct from those of other countries, where other clades and other clinical forms, like candidemia, are predominated. No case of candidemia was noted. Although fluconazole resistance was documented in 50% of the isolates, no resistance to echinocandins or amphotericin B was noted.

**Conclusion:**

This review highlighted the following notable points: (a) *C. auris* infection is a challenging condition which is usually misdiagnosed or diagnosed untimely; (b) the clinical table and cladal distribution of *C. auris* in Iran is different from those of other countries; (c) cases with *C. auris* may provide negative culture, underscoring the importance of using specific direct polymerase chain reaction to rule out or confirm *C. auris*, particularly in cases with chronic otomycosis; (d) ongoing patient follow-up and antifungal susceptibility testing in patients who do not respond to antifungal drug are recommended; (e) reliance on non-specific, insensitive, and time-consuming approaches, such as culture, may not only postpone the detection, but also lead to misidentification of *C. auris* in settings where molds are predominated.

## Introduction

Candidiasis represents the most prevalent fungal infection worldwide, encompassing both superficial and invasive forms, with candidemia posing particularly high mortality risks among intensive care unit (ICU) patients [1]. Current global estimates suggest that approximately 400,000 candidemia cases occur annually, predominantly reported from developed countries [2, 3]. While *Candida albicans* remains the primary etiological agent across most clinical settings, emerging epidemiological data have revealed a concerning shift toward non-*albicans Candida* species demonstrating reduced susceptibility to first-line antifungals, including fluconazole (the therapeutic mainstay in many regions) and echinocandins [3].

First identified in 2009 from an external ear canal specimen in Japan, *Candida auris* (Metchnikowiaceae, *Candida haemulonii* complex) has rapidly emerged as a concerning multidrug-resistant (MDR) nosocomial pathogen, demonstrating intrinsic resistance to fluconazole and variable susceptibility to other azoles, amphotericin B (AMB), and echinocandins [3, 4]. Within years of its discovery, this problematic yeast has spread globally, with both invasive and superficial infections reported in over 50 countries, characterized by frequent treatment failures due to high fluconazole resistance rates and documented clonal transmission within and between healthcare facilities [5].

Whole genome sequencing (WGS) analyses have revealed nearly identical *C. auris* isolates among patients within the same hospital in New Jersey, USA, strongly suggesting clonal outbreak transmission [3]. These findings underscore the critical need for both improved diagnostic methods to accurately assess disease burden and stringent infection control measures to prevent transmission [3, 4, 6]. Current diagnostic limitations, particularly the inability of conventional biochemical methods to reliably identify *C. auris*, contribute to significant underdiagnosis and underestimation of infections [3-6]. Furthermore, existing studies predominantly focus on culture-positive cases, overlooking culture-negative infections that escape routine detection, thereby distorting true epidemiological understanding [2, 5].

Although initial reports from Japan and South Korea have identified ear discharge as the primary source of *C. auris* isolates, systematic reviews have indicated that blood has become the predominant specimen type globally, with *C. auris* altering candidemia epidemiology in several countries and even surpassing *C. albicans* prevalence in some South African and Indian healthcare centers [7, 8]. In contrast, studies from Iran have reported no bloodstream infections; instead, external ear infections were identified as the most common clinical manifestation, accounting for 83% of cases [6, 9-13]. Genomic analyses have revealed that most Iranian isolates belong to the distinct clade V [9, 10, 13], except for two cases (a cerebrospinal fluid [CSF] isolate from Tehran and a case from Bushehr) classified as clade I [12, 14].

Besides six culture-confirmed cases from Iran, our prior study detected four additional cases through species-specific polymerase chain reaction (PCR). The present case series aimed to represent the first comprehensive clinical characterization of four *C. auris* cases (both culture-positive and negative) identified among 1,029 isolates/specimens using *C. auris*-specific PCR, complemented by a systematic review of all Iranian cases to provide a complete epidemiological context.

## Case Presentation

### Case 1

A 46-year-old man, who had lost his left ear hearing after middle ear surgery in childhood, presented to the Otorhinolaryngology Clinic in Al-Zahra Hospital, Isfahan, Iran, in April 2018 while manifesting severe ear pain on the left side. He had a history of numerous relapses with pruritus, tinnitus, otorrhea, and auricular inflammation during this period, and had visited an otorhinolaryngology clinic several times for cleaning of the ear canal. However, the symptom improvement was only temporary. Therefore, the patient was reassessed by an otolaryngologist with the suspicion of infectious otitis based on associated clinical symptoms. The otoscopic examination was performed, which revealed pus in the left external auditory canal. An ear aspiration was collected, and the specimen was sent for mycological examination. Microscopy examination of the specimen using both 10% potassium hydroxide (KOH; Merck, Germany) and Giemsa stain (Sigma-Aldrich, Germany) demonstrated the presence of septate hyphae and a few yeast cells. Moreover, on Sabouraud dextrose agar (SDA) plates containing chloramphenicol (50 mg/L) (Biolife, Italy), growth of Aspergillus flavus was evidenced, whose identity was further confirmed by PCR and sequence analysis of the beta-tubulin gene [15]. The patient was managed on an outpatient basis through frequent cleaning of the ear canal and administration of 10% canazole solution. At the last follow-up, the symptoms were found to be diminished using this therapeutic approach; however, complete recovery had not been achieved at the last visit, and the patient did not return thereafter.

One year later, the ear aspiration specimen of the patient, which had been stored at -20 °C, was included in a surveillance study screening for detection of *C. auris* by means of direct molecular techniques [6]. The specimen was subjected to manual DNA extraction using phenol-chloroform extraction after tissue digestion with a lysis buffer (Pouyagene, Iran) [16]. A direct *C. auris*-specific PCR was performed using the previously described primer pairs targeting a 250 bp fragment of the internal transcribed spacer (ITS)-rDNA region of *C. auris* (F: 5’-ATTTTGCATACACACTGATTTGG-3’; R: 5’-AATCTTCGCGGTGGCGTT-3’; Pishgam, Iran), which yielded a positive PCR. The amplicon was further confirmed as *C. auris* by sequencing. The sequence was deposited in the GenBank (accession no. OK595359.1) [6]. Although the specimen was cultured again, no yeast colonies were noted, and only *A. flavus* grew. The patient was asked about any international trips, and a history of traveling to Syria and Iraq was reported, but he never had a pet.

Furthermore, when the patient was telecommunicated in January 2021, during the Coronavirus disease 2019 (COVID-19) pandemic, he was still suffering from severe itching, otalgia, tinnitus, and otorrhea in his left ear despite receiving the antifungal therapy. Therefore, he was recalled for re-admission to the Otorhinolaryngology Clinic to be reassessed and managed based on his *C. auris* PCR-positive result. On otoscopic examination, the left external ear canal was markedly painful, congested, and filled with purulent cheesy discharge.

Initially, a diagnosis of chronic otitis externa was considered. Samples (ear aspirations and ear swab specimens) were collected from both ears. which indicated the presence of hyphae and a few yeast cells only in the left ear on direct examination using 10% KOH. The samples were simultaneously cultured on SDA supplemented with chloramphenicol (50 mg/L), CHROMagar *Candida* (BioMérieux, France), and incubated at 35 °C, as well as Salty SDA supplemented with chloramphenicol, 10% NaCl (wt/vol) (Merck, Germany), and D-Mannitol (as carbon sources) (SIGMA, Germany), which was incubated at 40 °C for a maximum of two weeks [11]. The growth of filamentous fungi (A. flavus) from the left ear discharge was observed one more time on those media (Figure 1-a). Moreover, a small number of yeast colonies grew on both SDA and CHROMagar *Candida*. Culture of the right ear discharge remained sterile.

**Figure 1:**
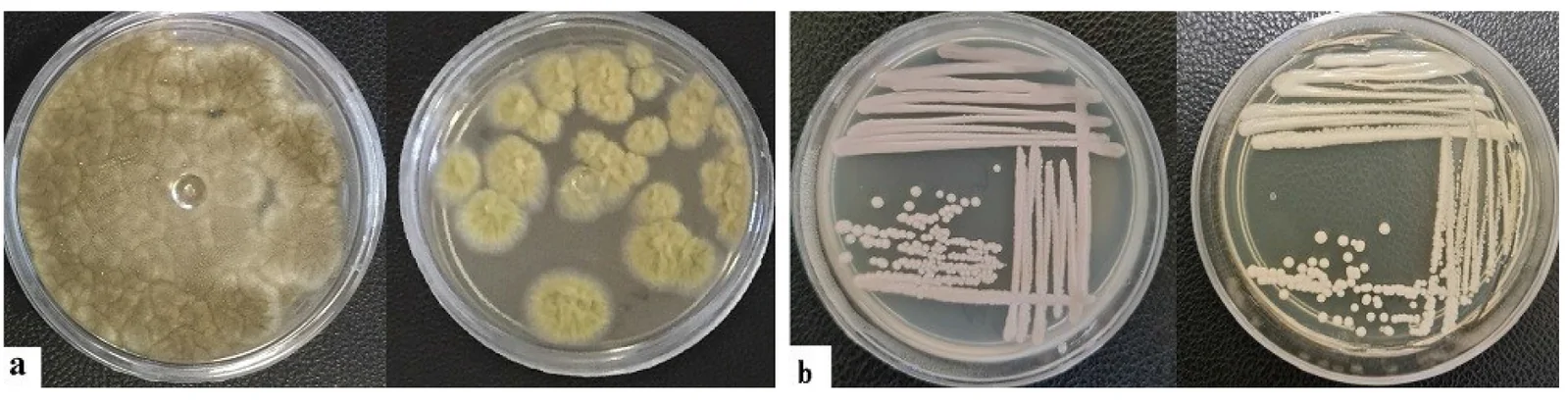
Morphological characteristics of pure colonies of *Aspergillus flavus* in the left ear on Sabouraud dextrose agar (SDA) upon 3 days of incubation at 35 °C as well as on Salt SDA upon 7 days of incubation at 40 °C; (a) pure culture of *Candida auris* on CHROMagar *Candida* (pink color) upon 3 days of incubation at 35 °C (left), and cream-colored colonies on SDA upon 3 days of incubation at 35 °C (right) (b).

As described above, the fresh left ear specimen was subjected to manual DNA extraction, using phenol-chloroform extraction, followed by the *C. auris*-specific PCR method [11], which yielded a negative result. Additionally, pan-fungal PCR was performed using the primer pairs ITS1 and ITS4 (Pishgam, Iran). The amplicon, which was sequenced, showed 99% similarity with the sequence of *A. flavus* deposited in NCBI BLAST (https://www.blast.ncbi.nlm.nih.gov/Blast.cgi).

To identify the yeast isolate, the colony was recultured on SDA medium, and the ITS region was amplified using the PCR-restriction fragment length polymorphism method [17]. The yeast was identified as *C. albicans*, and it was noteworthy that no further trace of *C. auris* was documented. However, as the symptoms were still presenting, the ear canal was cleaned, 1% clotrimazole ear drops were administered for two months, and regular visits were planned until all symptoms were resolved.

At the last follow-up (10 months later; on 8 December 2021), after the secondary drainage, much of the auricular edema had subsided, the erythema had entirely disappeared, and the patient had no further pain. The physician prescribed Oticept (hydrocortisone/acetic acid) twice a day for possible itching.

### Case 2

In April 2018, a 37-year-old housewife referred to the Otorhinolaryngology clinic in Al-Zahra Hospital, Isfahan, Iran, chiefly complaining of a sudden onset of pain in the left ear, and a history of hearing impairment, itching, and otorrhea. The patient had experienced chronic ear drainage with persistent recurrent infection despite aggressive outpatient topical treatment. On the initial otoscopic examination, a diagnosis of central tympanic membrane perforation was made, and the patient was taken to surgery for left tympanoplasty. The possibility of fungal infection in the ear canal was noticed. After the left tympanoplasty, the patient was advised to follow up outpatient care unit for further clinical evaluation. The ear canal debris and exudates collected during the surgery were submitted to the clinical mycology laboratory for mycological assessment. On direct KOH examination of the discharge, branching septate fungal hyphae and a small number of yeast cells were observed, and culture yielded mold colonies (black aspergilli). Precise morphological identification at the species level was not feasible. Therefore, the isolate was subjected to molecular identification and sequence analysis of the β-tubulin gene, which was identified as *Aspergillus* section *Nigri* [15]. As a result, antifungal drops (1% clotrimazole ear drops) were administered.

Similar to the previous case, one year later, the conserved ear aspiration specimen was subjected to a manual DNA extraction process by the phenol-chloroform method [16]. Moreover, the genomic DNA was tested with the aforementioned direct *C. auris*-specific PCR [6], which yielded a positive result. The PCR product was purified to be sequenced and confirmed. The result was analyzed using NCBI BLAST, and the amplicon was identified as *C. auris*. It should be mentioned that the sequence was deposited in the GenBank (accession no. OK595360.1) [6]. Culture of the ear aspiration specimen revealed the mold colonies (black aspergilli) again with no yeast colony.

In January 2021, at a follow-up call again due to being *C. auris* PCR-positive, the patient was asked about the associated symptoms. Tinnitus and pruritus were presented as the main complaints. The patient was referred to the Otorhinolaryngology Clinic. It must be noted that she had no history of traveling abroad. During the otoscopic examination, left tympanoplasty, and a small number of debris was noted at the right ear, with no purulent discharge; however, a wet swab specimen from both ears was provided for bacterial and fungal culture and microscopic examination. On direct examination using Gram stain, gram-positive cocci were observed, while no yeast cells were demonstrated. Culture of the ear discharge failed to reveal any causative organism. For itching, the patient was treated with Oticept (hydrocortisone/acetic acid) eardrop. After nine months of follow-up, she remained asymptomatic.

### Case 3

In July 2018, a 27-year-old male nurse presented to the Otorhinolaryngology Clinic with a several-day history of hearing impairment, otalgia, severe itching, and auricle inflammation in the left ear. He had no history of aural irrigation, ear surgery, or prolonged steroid use. The findings recorded through the otoscopic examination were suggestive of otomycosis (black residues in the outer ear canal). The tympanic membrane was found intact and undamaged. A swab specimen in a sterile condition was collected from the lesion for microbiological examination. During the otorhinolaryngology consultation, an ear cleaning suction was performed, and eardrop canazole 10% was prescribed twice daily for 15 days. Mycological cultures from the swab obtained from the outer ear canal showed growth of black aspergilli without any bacteria, while on direct examination, budding yeast cells and septate hyphae were observed together. Species identification of *Aspergillus* was attempted by sequencing the beta-tubulin gene, and the isolate was identified as *Aspergillus* section *Nigri* [15].

One year later, when the frozen ear aspiration specimen was screened for direct molecular detection of *C. auris* through the aforementioned *C. auris*-specific primers [6], a positive PCR was documented. Therefore, the PCR product was purified and sequenced, and the result was analyzed using NCBI BLAST, which confirmed the identity as *C. auris*. The sequence was deposited in the GenBank (accession no. OK595361.1) [6]. The ear discharge was cultured one more time, but only black aspergilli were found. The patient had no history of traveling abroad.

According to the PCR result, in January 2021, the patient was telecommunicated again, and no relapse was reported. Nonetheless, he refused to refer to the Otorhinolaryngology Clinic for re-evaluation; hence, the final outcome could not be ascertained.

### Case 4

A 33-year-old female presented in May 2017 with right otomycosis symptoms (pruritus, otalgia, hearing loss, and purulent otorrhea) and tympanic membrane rupture. Initial mycological analysis identified *A. flavus*, but antibacterial treatment (Myxacort) without antifungals led to treatment failure and worsened hearing post-tympanoplasty. Retrospective PCR of preserved specimens confirmed *C. auris* co-infection (GenBank OK595358.1). By February 2021, persistent bilateral symptoms prompted re-evaluation. Comprehensive diagnostics (KOH/gram staining, ITS-rDNA sequencing [MZ389242.1], matrix-assisted laser desorption ionization-time of flight mass spectrometry [Bruker, Bremen, Germany], WGS) confirmed *C. auris* clade V, with contamination ruled out through repeated sampling [11]. Antifungal susceptibility testing (AFST) revealed resistance to fluconazole/terbinafine, likely resistance to voriconazole/AMB, and susceptibility to echinocandins. Multidetector computed tomography (MDCT) showed left chronic otomastoiditis, prompting combined surgical intervention and clotrimazole therapy (Figure 2). This case represented the third reported *C. auris* clade V infection from chronic otitis media in Iran, with no travel history. Complete methodological and clinical details are available in the previous publication of the authors [[11].

**Figure 2:**
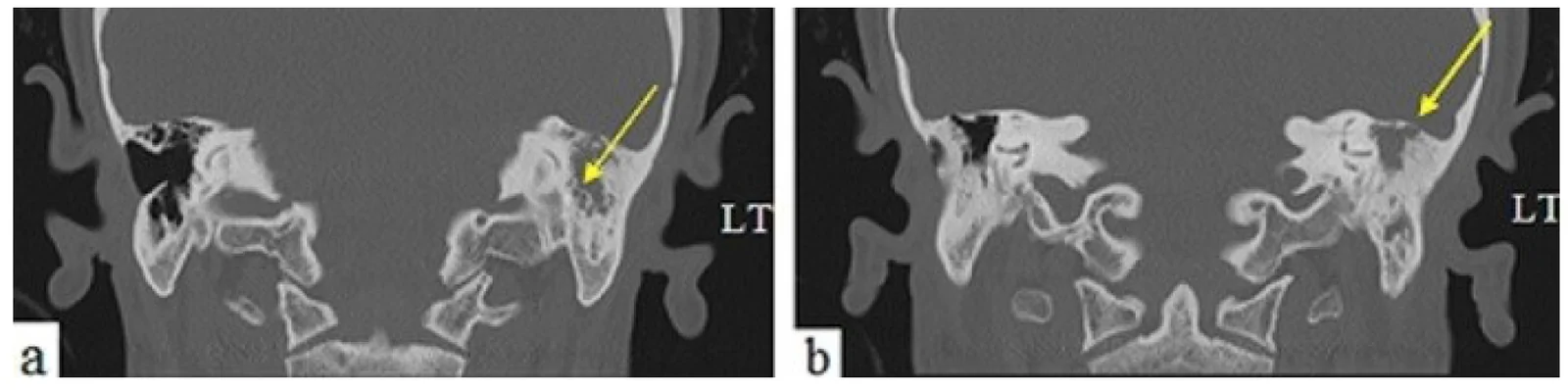
Multidetector computed tomography findings of otomastoiditis. After being treated with a long-term antifungal agent, the second follow-up of the patient was temporal bone computed tomography. Opacification of the left mastoid air cells seems to suggest mastoiditis. There is also opacification in the LT middle ear cavity in favor of infectious processes (arrow) (a-b), suggesting evidence of otomastoiditis in the LT side.

Nine months later (1 December 2021), during an in-person follow-up in the outpatient clinic, the otorhinolaryngology specialist noted no clinical response to the topical antifungal therapy, and the patient continued to report severe itching, tinnitus, and bilateral ear pain. However, the patient refused surgery due to her strong fear of losing the remaining hearing in her left ear, stemming from her previous experience with ear surgery on the right side. Otoscopic examination revealed pus in the left external auditory canal; moreover, edema of the surrounding tissues (symptoms of otomastoiditis), nasal obstruction, and discharge were observed, but no specimen was sent to the laboratory. Hearing of the patient was severely reduced, compared to the previous visit. The right ear had low hearing and needed hearing aids, and the left ear was impaired.

Therefore, the otorhinolaryngology specialist requested a tympanogram. Based on the tympanogram results, the right ear had moderate to profound hearing loss (mixed hearing loss) (speech recognition threshold: 60 dB; most comfortable loudness: 90 M; speech discrimination score: 92 %), and the left ear had moderate to severe hearing loss (mixed hearing loss) (speech recognition threshold: 60 dB; most comfortable loudness: 90 M; speech discrimination score: 100 %). The patient was also suffering from severe headaches and occasional vertigo. It should be noted that she had no immunocompromising conditions. Due to abnormalities, such as mastoiditis and left tympanic membrane perforation, emergency surgery for mastoidectomy, as well as left tympanoplasty, was strongly recommended. The patient was persuaded by the situation, and surgery was reserved for 45 days later (15 January 2022).

During hospitalization, the MDCT scan of the temporal bone again demonstrated chronic otitis media (COM) with granulation tissue formation on the left side. On the eleventh day after the surgical operation, a swab of both ears was sent for bacterial and fungal culture. Culture of the specimen collected from the left ear revealed the *Candida* species again, which was confirmed to be *C. auris* by molecular identification targeting the ITS rDNA region. Therefore, an infectious disease specialist was consulted for targeted antifungal therapy. The patient was admitted to the infectious disease ward. AFST of AMB, fluconazole, itraconazole, clotrimazole, micafungin, ketoconazole, terbinafine, and caspofungin against the isolate was requested, which was performed as mentioned above. The results are summarized in Table 1.

**Table 1:** Results of antifungal susceptibility testing (MIC, μg/ml) of different drugs against *Candida auris* isolated from case no. 4 at different sampling time points.

Date of sampling	Time	FLC	AMB	MICA	CASPO	ITC	VRC	5FC	ANID	CLO	KTO	TRB
First sampling 2021.2.6	24 h	>64	0.5	0.015	0.015	0.5	1	0.5	0.125			
Second sampling 2021.2.21	24 h	>64	1	0.5	0.015	0.5				16	0.125	>16
Third sampling 2022.3.15	24 h	>64	1	0. 125	>0.008	2				>16	0.5	>16

FLC: fluconazole, AMB: amphotericin B, MICA: micafungin, CASPO: caspofungin, ITC: itraconazole, VRC: voriconazole, 5FC: flucytosine, ANID: anidulafungin, CLO: clotrimazole, KTO: ketoconazole, TRB: terbinafine

Immunodeficiency-associated laboratory tests (IgG, IgM, IgA, CH50, C3 Complement, and C4 Complement tests) were requested. Moreover, probable brain involvement (brain MDCT scan) and abdominal and pelvic ultrasound were evaluated. Laboratory data, brain MDCT scan, and ultrasound showed no abnormalities. Intravenous liposomal AMB (L-AMB) was initiated (200 mg once a day). The patient showed desirable clinical improvement and biological tolerance to the antifungal therapy after 10 days.

After completion of treatment and during outpatient follow-up, a swab sample of both ears was sent for bacterial and fungal culture. Microscopic examination of the specimen did not reveal any microorganisms, and both fungal and bacterial cultures were negative. In the last in-person follow-up visit (on 20 June 2023), the patient was only suffering from mild ear pain; therefore, she was advised to use hearing aids and was recommended to visit the Otorhinolaryngology Clinic if any warning signs appeared.

## Materials and Methods

A systematic review was conducted to retrieve previously published cases of *C. auris* in Iran by searching electronic databases, including PubMed, Scopus, and Google Scholar for English-language reports (full text or abstract). The search strategy used the terms "C. auris" OR "Multi-antifungal drug resistance" combined with "Iran" OR "otomycosis" OR "candidiasis" OR "candidemia", followed by manual screening of references and bibliographies of relevant articles. Extracted data consisted of demographic characteristics, underlying diseases and predisposing factors, mycological and molecular evidence, clinical manifestations and disease type, as well as antifungal susceptibility, treatment, and outcome.

### Literature review

The database search yielded 24 articles (reviews or original/case reports) on *C. auris* from Iran, of which only six contained unique, non-redundant case reports meeting the inclusion criteria [9-14]. Including our four reported cases (although case number four was previously documented [11] but further followed here), the clinic-demographic characteristics of a total of nine *C. auris* cases in Iran were analyzed, as is illustrated in Figures 3 and 4.

**Figure 3:**
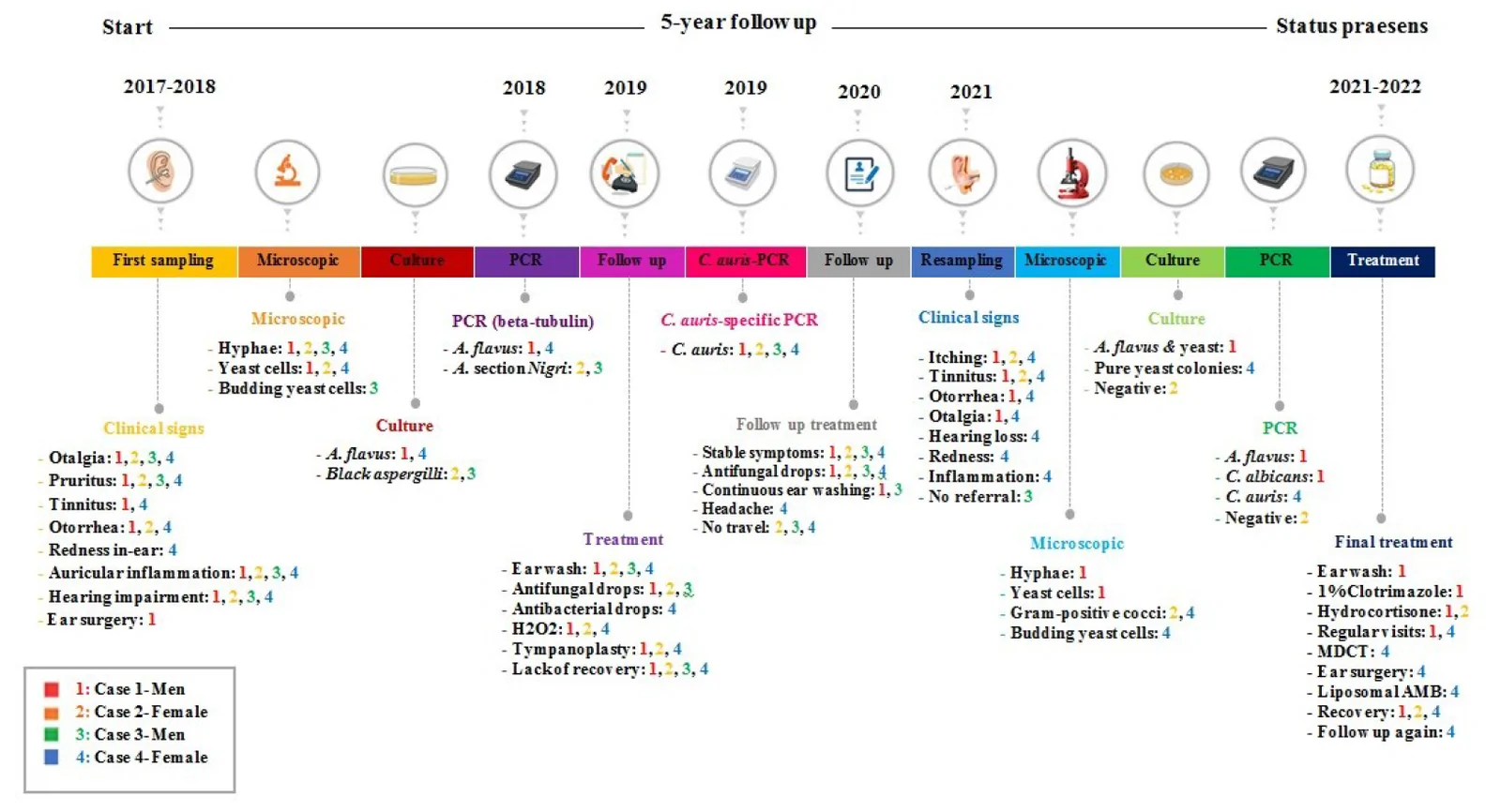
Timeline representing the course of four cases of *Candida auris* otomycosis in this study. This timeline depicts the date of the first and last sampling, clinical symptoms, the results of laboratory diagnosis, including microscopic, macroscopic, and molecular methods, and also the treatment of patients (all four cases presented in this study) during 5 years (2017-2022).

**Figure 4:**
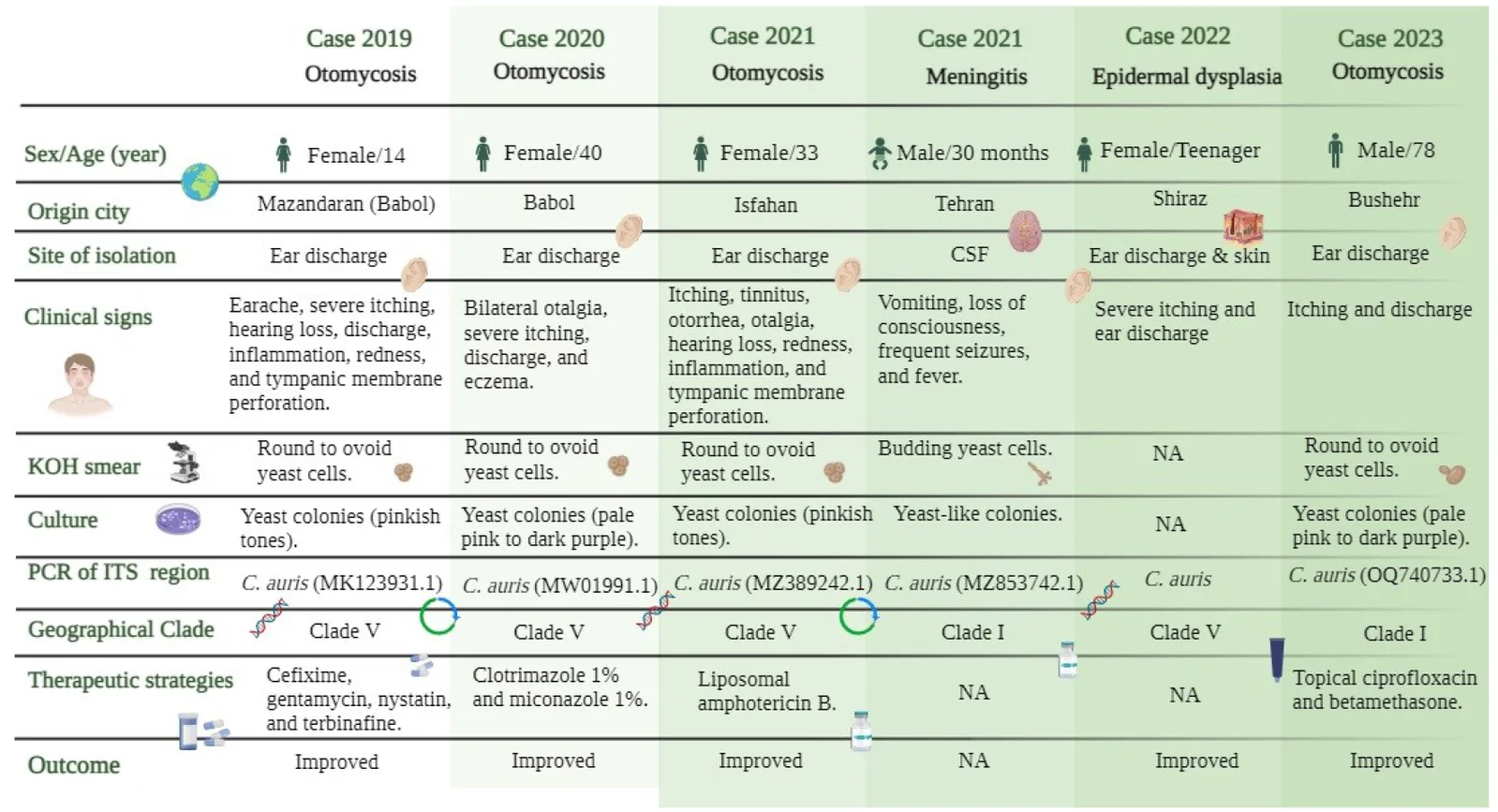
Patient characteristics and diagnostic details of six culture-proven Iranian *Candida auris* cases until 2023.

Median age of *C. auris* cases was 33 years (range: 30 months-78 years), with males (44.4%, 4/9) showing a lower prevalence than females. Underlying conditions were present in 33.3% of cases (3/9), including necrotizing pneumonia (CSF case), ichthyosis (Shiraz case), and type II diabetes mellitus (Bushehr case) [12-14]. Three clinical forms were identified, namely otitis (88.8%, 8/9 cases), meningitis (1 case), and epidermal dysplasia (Shiraz case, associated with otitis). No candidemia cases were reported, with ear involvement (otitis externa/middle ear infection) being the predominant manifestation.

The predominant predisposing factors for *C. auris* otomycosis cases (a total of 8) were moisture and frequent ear manipulation (62.5%, 5 cases), followed by diabetes mellitus (12.5%, 1 case) and ichthyosis (12.5%, 1 case) [6, 9-13]. For the single *C. auris* meningitis case, necrotizing pneumonia and prolonged ICU hospitalization in Pakistan were the sole identified risk factors [14].

All eight otomycosis patients (100%) presented with pruritus and otalgia, while 62.5% (5/8) exhibited auricular inflammation and hearing impairment. Ear involvement was unilateral left (50%, 3/6), unilateral right (16.6%, 1/6), or bilateral (33.3%, 2/6), with two cases (Shiraz and Bushehr) lacking laterality documentation. Imaging revealed mastoiditis in one patient (case 4 [2021]; otomycosis) and acute hydrocephalus in another (meningitis case), with one case requiring mastoidectomy. Tympanic membrane rupture was observed in 50% (4/8) of otomycosis cases, with three patients (all from the current study) undergoing tympanoplasty.

*Candida auris* cultures were positive in 66.6% (6/9) of cases, confirmed by colony-based molecular methods (sequencing or *C. auris*-specific PCR). Direct PCR identified *C. auris* in four cases (three culture-negative, one positive [case 4 [2021]; otomycosis]) [6], all from the current study, with no prior Iranian reports using this approach. Multilocus sequence typing (MLST) analysis showed clade V predominance (4/6 cases) versus clade I (2/6).

Antifungal resistance was identified in 4/6 culture-positive *C. auris* cases, while susceptibility testing was not performed on the three culture-negative cases. Fluconazole resistance predominated (50%, 3/6 isolates), followed by voriconazole (33%, 2/6), with no observed resistance to echinocandins or AMB.

Monotherapy (surgery or single antifungal agents) was used in 78% (7/9) of cases, with 43% (3/7) receiving antifungal treatment alone. Surgery alone was performed in 37.5% (3/8) of otomycosis cases, while combined antifungal-surgical intervention was used in 11.1% (1/9) of cases. One meningitis case received neither treatment. Combination antifungal therapy (≥2 agents) was employed in 57% (4/7) of cases. No fatalities occurred, although the treatment failure rate was 16.6% (1/6). Notably, three cases with initial treatment failure improved after proper *C. auris* identification and targeted therapy (all current study cases).

## Discussion

The emerging fungal pathogen *C. auris*, reported globally, causes superficial to invasive candidiasis with high treatment failure rates and significant mortality in immunocompromised patients [18]. The Centers for Disease Prevention and Control documented a dramatic increase in U.S. cases from 10 (2015) to 700 (2020), with mortality reaching 60% [19]. On October 26, 2022, the World Health Organization classified *C. auris* among the highest-priority life-threatening fungi alongside *Aspergillus fumigatus*, *C. albicans*, and *Cryptococcus neoformans* [19].

The rising incidence of fungal infections necessitates enhanced surveillance and laboratory capacity for early detection of MDR species, like *C. auris*, as emerging pathogens may be missed due to nonspecific symptoms and inadequate diagnostic methods. Despite the increase in *C. auris* reports in neighboring countries, Iran has documented only six cases (five otitis externa [9-13] and one meningitis [14]), suggesting significant underreporting. This underestimation stems from phenotype-based misidentification (e.g., biochemical tests confusing *C. auris* with other yeasts), limited molecular diagnostics (sequencing/specific primers), reliance on culture-based methods, and failure to consider PCR-positive/culture-negative cases [6].

This study details the clinical features of 4 *C. auris* cases (both culture-positive and negative) identified through *C. auris*-specific PCR screening of 1,029 yeast isolates/specimens, combined with a literature review of prior Iranian cases [6, 9-14]. Notably, our single-center, retrospective study performed in Isfahan (2019-2021) identified four cases (two annually), exceeding the three cases reported nationally in the preceding three years [9, 10, 14]. This increasing detection trend in Iran mirrors global patterns and likely reflects heightened clinical awareness, improved diagnostic capacity, and recognition of culture-negative cases through direct PCR, as demonstrated by 4/9 Iranian cases testing PCR-positive despite initial culture negativity [6].

This review revealed a distinctive clinical pattern of *C. auris* infections in Iran, predominantly manifesting as otomycosis (88.8%, 8/9 cases) with only one meningitis case (11.1%), in contrast with global reports where candidemia prevails as the dominant form [4]. While earlier studies have corroborated tropism of *C. auris* for the external ear canal [3, 20], its emergence has significantly altered candidemia epidemiology in some regions, even surpassing *C. albicans* prevalence in certain South African hospitals [8]. The documented potential for rapid nosocomial transmission through contaminated medical equipment is evidenced by outbreaks of genetically identical MDR strains in Indian healthcare facilities (2013-2014) and clustering of 6/9 Iranian cases (including our four cases plus two cases from Babol) [3]. This underscores the critical need for rigorous identification protocols, patient isolation, and enhanced sterilization/disinfection measures to curb potential outbreaks [3].

The present review revealed a female predominance in *C. auris* otomycosis cases (55.5%, 5/9), consistent with South Korean data reporting 54.4% female cases [21]. While otomycosis typically shows right-ear predominance in unilateral cases [22], the present study demonstrated left-ear involvement, right-ear involvement, and bilateral infection in 37.5% (3/8), 12.5% (1/8), and 25% (2/8) of cases with otitis media (Shiraz and Bushehr cases lacked laterality data). Bilateral involvement may result from cross-contamination during ear swab procedures.

This study found *C. auris* culture positivity in 66.6% (6/9) of Iranian cases, with direct PCR detecting four additional cases (three culture-negative and one positive) from our cohort, later confirmed by sequencing. We developed a species-specific PCR to identify *C. auris* directly from clinical samples, bypassing culture limitations [6]. Notably, all patients had concurrent otitis externa due to *Aspergillus*, where mold overgrowth often masks yeast presence [6]. Culture-independent methods prove more effective for detecting rare fungi in mold-dominated specimens, like ear samples [6, 23, 24]. While PCR detects residual or nonviable *C. auris* DNA (75% PCR-positive/culture-negative cases), results require careful interpretation [6]. This approach prevents missed diagnoses, as demonstrated by one case (case four [2021]; otomycosis) where PCR detection revealed persistent *C. auris* infection despite prior antifungal therapy, ultimately causing COM and otomastoiditis after 5 years [11]. Diagnostic delays due to nonspecific presentations often lead to complications, like tympanic membrane perforation (50% in our otomycosis cases) and otomastoiditis [11]. Given the rising *C. auris* otomycosis incidence, we recommend rapid screening methods, such as specific PCR (high negative predictive value and quick turnaround) [6, 22]. Optimal treatment combines surgical debridement with prolonged antifungal therapy [11, 25].

While most *C. auris* cases in Iran involved superficial infections with expected low mortality, our otomycosis cases presented therapeutic challenges due to high antifungal resistance rates (83.3% in culture-positive cases), necessitating multiple surgical debridements and treatment modifications. Although invasive *C. auris* infections (particularly candidemia) show 30-60% mortality rates [3, 26], antifungal therapy remains crucial for patient outcomes [3, 27]. Echinocandins, demonstrating the lowest resistance rates (2-8%), are first-line treatments [28], while SCY-078 shows promise for MDR isolates (≤4% resistance) through its anti-biofilm activity [3]. In our cohort, only one patient (case four) received L-AMB with surgery for otomastoiditis and no fluconazole use.

This study revealed no echinocandin, L-AMB, or MDR resistance among Iranian *C. auris* isolates (6/9 culture-positive cases), although 50% (3/6) demonstrated fluconazole resistance (MIC>32->64 μg/mL) and 33% (2/6) showed voriconazole resistance (MIC≥1 μg/mL). *Candida auris* exhibits intrinsic fluconazole resistance (>90% isolates MIC>2 μg/mL) and intermediate voriconazole susceptibility (50% isolates) [3], consistent with the findings of Kim et al. [3], who reported ear isolates with fluconazole MICs of 64-128 μg/mL [20, 25]. While 35% of global isolates show AMB resistance and 2-8% echinocandin resistance [3, 29], newer triazoles (posaconazole/isavuconazole) demonstrate promising *in vitro* activity despite undefined breakpoints [29]. It is noteworthy that in the present study, AFST was unavailable for three culture-negative cases, prompting empirical treatment in two patients (Cases one and two). Tympanoplasty was performed adjunctively in 37.5% (3/8) of cases to restore tympanic membrane integrity and hearing. Given the resistance profile of *C. auris*, combination antifungal therapy is essential upon suspicion to prevent treatment failure [30], underscoring the need for prompt diagnosis and multidrug regimens [31].

Lockhart et al. [29] identified four genetically distinct *C. auris* clades (South Asian/I, East Asian/II, African/III, South American/IV) through WGS, each showing geographic specificity with tens of thousands of single-nucleotide polymorphism differences [29]. A potential fifth clade was recently found in an Iranian ear swab isolate, genetically distinct (hundreds of thousands of single-nucleotide polymorphisms) from known clades despite pathological similarity to clade II, suggesting local emergence rather than recent introduction [32]. Clade-specific resistance patterns include > 90% fluconazole resistance in clades I and III, ~50% in clade IV, with polyene/echinocandin resistance also reported, while clade II remains largely susceptible [5]. Clinically, clades I, III, and IV associate with nosocomial outbreaks and invasive infections, whereas clades II and V primarily cause ear colonization/infections [33].

The MLST analysis of Iranian *C. auris* cases (6/9 culture-positive) revealed clade V (Iranian Clade) in four cases [9-11, 13] and clade I (South Asian) in two cases [12, 14]. Five patients had no international travel history, supporting local emergence of clade V, while one meningitis case (clade I) with prior hospitalization in Pakistan [14] reflected known geographic clustering patterns [3, 33]. Phylogenetic analysis of ITS rDNA showed Iranian isolates cluster closely with each other and a Japanese ear isolate [27, 29, 33]. Notably, the CSF isolate (clade I) demonstrated genetic relatedness to Iranian/Japanese ear isolates despite the Pakistan travel history of the patient [14], suggesting epidemiological links between these strains (Figure 5).

**Figure 5:**
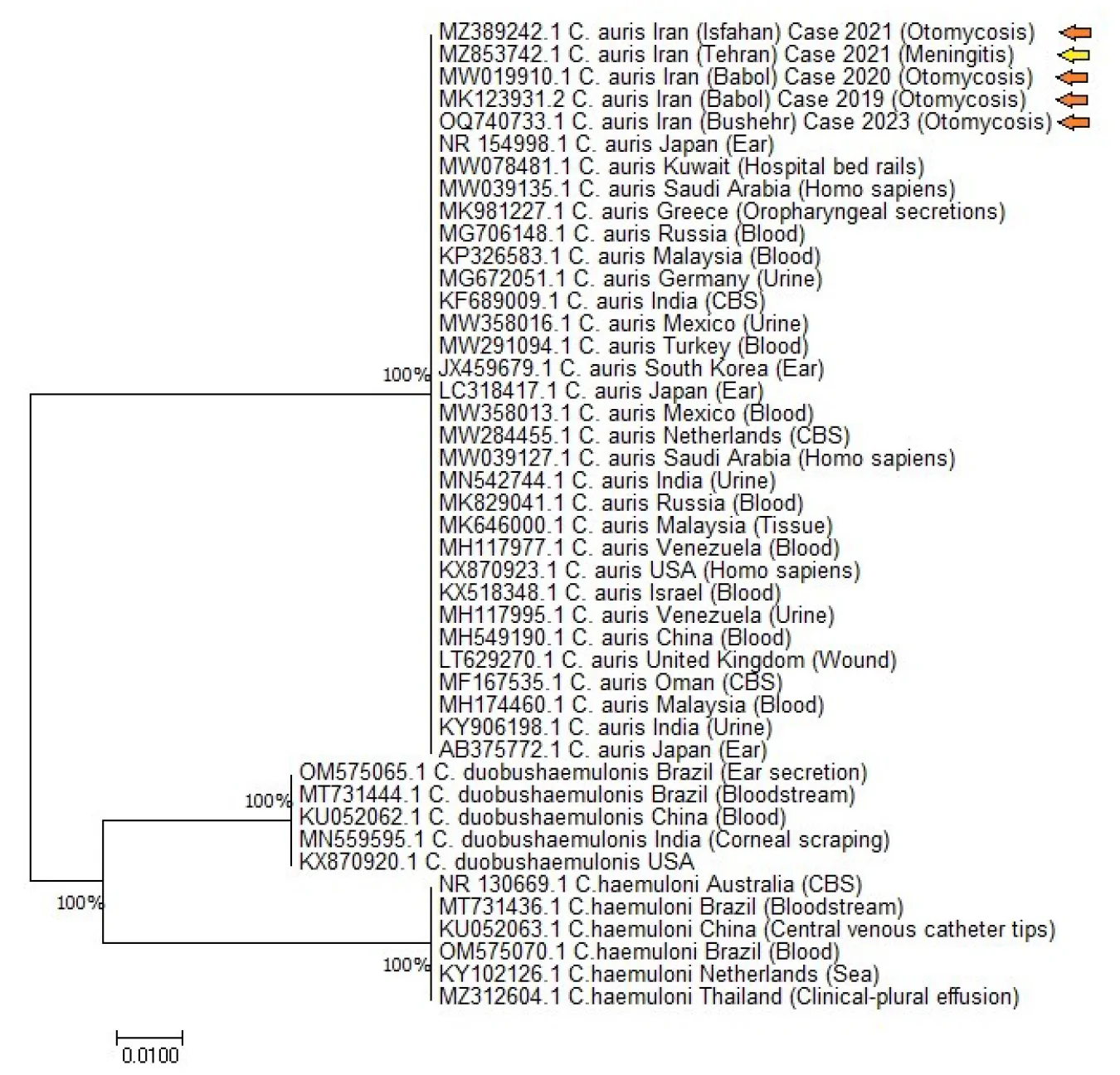
Dendrograms of *C. auris* strains. Trimmed internal transcribed spacer 1/4 (ITS 1 and ITS4) were aligned using MEGA7 software. Arrows indicate *C. auris* strains (positive culture; 5/9) identified in Iran (GenBank accession numbers MK123931.2, MW019910.1, MZ389242.1, OQ740733.1, and MZ853742.1).

The literature review identified limited reports of *C. auris* isolation in CSF. In this regard, Noginskiy et al. [34] documented a case of *Streptococcus pneumoniae* meningitis complicated by *C. auris* fungemia in a multiple myeloma patient. These findings suggest immunodeficiency and prolonged hospitalization as key risk factors for developing life-threatening *C. auris* infections.

## Conclusion

This comprehensive analysis revealed six critical findings about *C. auris* infections: (1) frequent misdiagnosis and delayed treatment; (2) distinct clinical presentations in Iran (predominantly otomycosis from clade V) versus global patterns (candidemia from other clades); (3) high culture-negative rates necessitating PCR for accurate detection; (4) essential role of antifungal susceptibility testing for treatment-refractory cases; (5) limitations of culture-based methods in mold-dominated specimens; and (6) high resistance rates of fluconazole (requiring echinocandin-first therapy). It should be noted that early pathogen identification remains crucial for optimal outcomes.

## References

[1] Mayer FL, Wilson D, Hube B (2013). Candida albicans pathogenicity mechanisms. Virulence.

[2] Kord M, Salehi M, Hashemi SJ, Abdollahi A, Alijani N, Maleki A (2022). Clinical, epidemiological, and mycological features of patients with candidemia: Experience in two tertiary referral centers in Iran. Curr Med Mycol.

[3] Chowdhary A, Sharma C, Meis JF (2017). Candida auris: A rapidly emerging cause of hospital-acquired multidrug-resistant fungal infections globally. PLoS Pathog.

[4] Jeffery-Smith A, Taori SK, Schelenz S, Jeffery K, Johnson EM, Borman A (2018). Candida auris: a Review of the Literature. Clin Microbiol Rev.

[5] Spruijtenburg B, Badali H, Abastabar M, Mirhendi H, Khodavaisy S, Sharifisooraki J (2022). Confirmation of fifth Candida auris clade by whole genome sequencing. Emerg Microbes Infect.

[6] Safari F, Madani M, Kheirollahi M, Mirhendi H (2022). Molecular investigation of the incidence of Candida auris infections at selected hospitals in Iran. Mycoses.

[7] Rudramurthy SM, Chakrabarti A, Paul RA, Sood P, Kaur H, Capoor MR (2017). Candida auris candidaemia in Indian ICUs: analysis of risk factors. J Antimicrob Chemother.

[8] van Schalkwyk E, Mpembe RS, Thomas J, Shuping L, Ismail H, Lowman W (2019). Epidemiologic Shift in Candidemia Driven by Candida auris, South Africa, 2016-2017(1). Emerg Infect Dis.

[9] Armaki MT, Omran SM, Kiakojuri K, Khojasteh S, Jafarzadeh J, Tavakoli M (2021). First fluconazole-resistant Candida auris isolated from fungal otitis in Iran. Curr Medi Mycol.

[10] Abastabar M, Haghani I, Ahangarkani F, Rezai MS, Taghizadeh Armaki M, Roodgari S (2019). Candida auris otomycosis in Iran and review of recent literature. Mycoses.

[11] Safari F, Madani M, Badali H, Kargoshaie AA, Fakhim H, Kheirollahi M (2022). A Chronic Autochthonous Fifth Clade Case of Candida auris Otomycosis in Iran. Mycopathologia.

[12] Ahmadi B, Naeimi B, Ahmadipour MJ, Morovati H, de Groot T, Spruijtenburg B (2023). An Autochthonous Susceptible Candida auris Clade I Otomycosis Case in Iran. J Fungi (Basel)..

[13] Park J, Youssefian L, Khodavaisy S, Palizban F, Tavasoli A, Kalamati E (2022). 493 Chronic mucocutaneous candidiasis due to Candida auris and non-albicans Candida species in a family with a mild TP63-associated ectodermal dysplasia. J Investigative Dermatol.

[14] Mirhendi H, Charsizadeh A, Aboutalebian S, Mohammadpour M, Nikmanesh B, de Groot T (2022). South Asian (Clade I) Candida auris meningitis in a paediatric patient in Iran with a review of the literature. Mycoses.

[15] Kiakojuri K, Mahdavi Omran S, Roodgari S, Taghizadeh Armaki M, Hedayati MT, Shokohi T (2021). Molecular Identification and Antifungal Susceptibility of Yeasts and Molds Isolated from Patients with Otomycosis. Mycopathologia.

[16] Ahmadi B, Mirhendi H, Makimura K, de Hoog GS, Shidfar MR, Nouripour-Sisakht S (2016). Phylogenetic analysis of dermatophyte species using DNA sequence polymorphism in calmodulin gene. Med Mycol.

[17] Mirhendi H, Makimura K, Khoramizadeh M, Yamaguchi H (2006). A one-enzyme PCR-RFLP assay for identification of six medically important Candida species. Nihon Ishinkin Gakkai Zasshi.

[18] Osei Sekyere J (2018). Candida auris: A systematic review and meta-analysis of current updates on an emerging multidrug-resistant pathogen. Microbiologyopen.

[19] Mosbergen D (2022). Fatal Fungi Threaten Global Health, WHO Says.

[20] Kim MN, Shin JH, Sung H, Lee K, Kim EC, Ryoo N (2009). Candida haemulonii and closely related species at 5 university hospitals in Korea: identification, antifungal susceptibility, and clinical features. Clin Infect Dis.

[21] Jung J, Kim MJ, Kim JY, Lee JY, Kwak SH, Hong MJ (2020). Candida auris colonization or infection of the ear: A single-center study in South Korea from 2016 to 2018. Med Mycol.

[22] Viswanatha B, Sumatha D, Vijayashree MS (2012). Otomycosis in immunocompetent and immunocompromised patients: comparative study and literature review. Ear Nose Throat J.

[23] Sexton DJ, Kordalewska M, Bentz ML, Welsh RM, Perlin DS, Litvintseva AP (2018). Direct Detection of Emergent Fungal Pathogen Candida auris in Clinical Skin Swabs by SYBR Green-Based Quantitative PCR Assay. J Clin Microbiol.

[24] Mulet Bayona JV, Salvador Garcia C, Tormo Palop N, Gimeno Cardona C (2021). Validation and implementation of a commercial real-time PCR assay for direct detection of Candida auris from surveillance samples. Mycoses.

[25] Choi HI, An J, Hwang JJ, Moon SY, Son JS (2017). Otomastoiditis caused by Candida auris: Case report and literature review. Mycoses.

[26] Spivak ES, Hanson KE (2018). Candida auris: an Emerging Fungal Pathogen. J Clin Microbiol.

[27] Kean R, Brown J, Gulmez D, Ware A, Ramage G (2020). Candida auris: A Decade of Understanding of an Enigmatic Pathogenic Yeast. J Fungi (Basel)..

[28] Lone SA, Ahmad A (2019). Candida auris-the growing menace to global health. Mycoses.

[29] Lockhart SR, Etienne KA, Vallabhaneni S, Farooqi J, Chowdhary A, Govender NP (2017). Simultaneous Emergence of Multidrug-Resistant Candida auris on 3 Continents Confirmed by Whole-Genome Sequencing and Epidemiological Analyses. Clin Infect Dis.

[30] Alfouzan W, Dhar R, Albarrag A, Al-Abdely H (2019). The emerging pathogen Candida auris: A focus on the Middle-Eastern countries. J Infect Public Health.

[31] Schwartz I, Hammond G (2017). Outbreak Detection: First reported case of multidrug-resistant Candida auris in Canada. Can Commun Dis Rep.

[32] Chow NA, de Groot T, Badali H, Abastabar M, Chiller TM, Meis JF (2019). Potential Fifth Clade of Candida auris, Iran, 2018. Emerg Infect Dis.

[33] Ashkenazi-Hoffnung L, Rosenberg Danziger C (2023). Navigating the New Reality: A Review of the Epidemiological, Clinical, and Microbiological Characteristics of Candida auris, with a Focus on Children. J Fungi (Basel)..

[34] Noginskiy I, Samra A, Nielsen K, Kalavar MR (2018). A Case of Multiple Myeloma Presenting as Streptococcus pneumoniae Meningitis with Candida auris Fungemia. Case Rep Oncol.

